# Bioinformatic analysis identifies HPV-related tumor microenvironment remodeling prognostic biomarkers in head and neck squamous cell carcinoma

**DOI:** 10.3389/fcimb.2022.1007950

**Published:** 2022-11-08

**Authors:** Qimin Zhou, Ouyang Yuan, Hongtu Cui, Tao Hu, Gary Guishan Xiao, Jiao Wei, Honglei Zhang, Chengjun Wu

**Affiliations:** ^1^ Department of Plastic and Reconstructive Surgery, Shanghai Ninth People’s Hospital, Shanghai Jiao Tong University School of Medicine, Shanghai, China; ^2^ Department of Oncology, Shanghai Ninth People’s Hospital, Shanghai Jiao Tong University School of Medicine, Shanghai, China; ^3^ School of Biomedical Engineering, Dalian University of Technology, Dalian, China; ^4^ School of Pharmaceutical Science and Technology, Dalian University of Technology, Dalian, China; ^5^ Department of Otolaryngology Head and Neck Surgery, Air Force Medical Centre, People's Liberation Army (PLA), Beijing, China

**Keywords:** head and neck squamous cell carcinoma, HPV, biomarkers, tumor microenvironment, immune evasion, immunotherapy

## Abstract

Head and neck squamous cell carcinomas (HNSCCs) are highly aggressive tumors with rapid progression and poor prognosis. Human papillomavirus (HPV) infection has been identified as one of the most important carcinogens for HNSCC. As an early event in HNSCC, infection with HPV leads to altered immune profiles in the tumor microenvironment (TME). The TME plays a key role in the progression and transformation of HNSCC. However, the TME in HNSCC is a complex and heterogeneous mix of tumor cells, fibroblasts, different types of infiltrating immune cells, and extracellular matrix. Biomarkers relevant to the TME, and the biological role of these biomarkers, remain poorly understood. To this end, we performed comprehensive analysis of the RNA sequencing (RNA-Seq) data from tumor tissue of 502 patients with HNSCC and healthy tissue of 44 control samples. In total, we identified 4,237 differentially expressed genes, including 2,062 upregulated and 2,175 downregulated genes. Further in-depth bioinformatic analysis suggested 19 HNSCC tumor tissue-specific genes. In the subsequent analysis, we focused on the biomarker candidates shown to be significantly associated with unfavorable patient survival: *ITGA5*, *PLAU*, *PLAUR*, *SERPINE1*, *TGFB1*, and *VEGFC*. We found that the expression of these genes was negatively regulated by DNA methylation. Strikingly, all of these potential biomarkers are profoundly involved in the activation of the epithelial–mesenchymal transition (EMT) pathway in HNSCCs. In addition, these targets were found to be positively correlated with the immune invasion levels of CD4^+^ T cells, macrophages, neutrophils, and dendritic cells, but negatively correlated with B-cell infiltration and CD8^+^ T-cell invasion. Notably, our data showed that the expression levels of *ITGA5*, *PLAU*, *PLAUR*, *SERPINE1*, and *TGFB1* were significantly overexpressed in HPV-positive HNSCCs compared to normal controls, indicating the potential role of these biomarkers as transformation and/or malignant progression markers for HNSCCs in patients with HPV infection. Taken together, the results of our study propose *ITGA5*, *PLAU*, *PLAUR*, *SERPINE1*, and *TGFB1* as potential prognostic biomarkers for HNSCCs, which might be involved in the HPV-related TME remodeling of HNSCC. Our findings provide important implications for the development and/or improvement of patient stratification and customized immunotherapies in HNSCC.

## Introduction

Head and neck cancers rank as the sixth most frequent malignancy worldwide, with more than 90% of this disease comprising head and neck squamous cell carcinoma (HNSCC), including different squamous epithelium-derived tumors of the oral cavity, oropharynx, larynx, and hypopharynx (Schmitz and Machiels, 2010; Bray et al., 2018; Chow, 2020). Recent statistics have pointed out that around 890,000 new cases and more than 450,000 deaths from HNSCC occur worldwide per year, accounting for 1%–2% of all malignant tumors, which is projected to exceed 30% by the year 2030 (Ferlay et al., 2019).

Aside from alcohol consumption and smoking, infection with human papillomavirus (HPV) is an increasingly common risk factor for HNSCC. HPV infection is associated with most oropharyngeal cancers (>70%) and a small minority of cancers at other anatomical sites in the head and neck (Leemans et al., 2018; Johnson et al., 2020; Lechner et al., 2022). Depending on the HPV infection status, HNSCCs are subdivided into HPV-negative (HPV^−^) and HPV-positive (HPV^+^) types, which have been recognized as distinct entities due to these tumors displaying a plethora of molecular and clinicopathological differences (Leemans et al., 2018). According to the latest reports, HPV^+^ HNSCCs represent at least 25% of the HNSCC cases worldwide (Lechner et al., 2022). Alarmingly, the global frequency of HPV^+^ HNSCCs has increased dramatically during the last few decades (D'Souza et al., 2007). For instance, the epidemic proportions of HPV^+^ oropharyngeal cancers have risen from 7.2% (1990–1994) to 32.7% (2010–2012), with a similar epidemic boost of HPV^+^ tonsillar cancers from 25% (1993–1999) to 62% (2006–2011) also being reported (D'Souza et al., 2007; Nichols et al., 2013; Nichols et al., 2013).

Unfortunately, despite previous studies suggesting an indicative role of the HPV status in the prognosis of HNSCC patients, its eligibility for guiding specific treatments is, to date, unclear (Sun et al., 2021). Therefore, regardless of HPV^+^ and HPV^−^ HNSCC patients presenting remarkable differences in characterization, they are still provided the same therapeutic strategies (Ghiani and Chiocca, 2022). Adopting a combination of surgery, chemotherapy, and radiotherapy for patients with different HPV status has resulted in unignorable side effects, as well as a tremendous economic burden to society (Schaaij-Visser et al., 2010; Braakhuis et al., 2012). As a result, a 5-year survival rate of only a less than 50% has been achieved (Schaaij-Visser et al., 2010; Braakhuis et al., 2012). Thus, it is a necessary and urgent requirement to identify new biomarkers for HNSCC that would benefit patient stratification and survival improvement.

During the past few years, immunotherapies have dramatically changed the treatment algorithm of various cancer types (Hamid et al., 2013; Powles et al., 2020). Recently, for recurrent and metastatic HNSCC patients, programmed death-1 (PD-1) targeted checkpoint inhibitors (CPIs) have been approved as the first- and second-line approaches (Ferris et al., 2016; Burtness et al., 2019; Cohen et al., 2019). However, the treatment effects of CPIs rely mainly on the infiltration, reinvigoration, and activation of tumor-oriented antigen-specific T cells (Gubin et al., 2014), and HPV-infected malignant cells can change the tumor microenvironment (TME), affecting antigen presentation and immune cell infiltration (particularly the CD4^+^/CD8^+^ T-cell ratio), which have a significant impact on HNSCC immunotherapy (Lechner et al., 2022). Unfortunately, only 15%–20% of patients with HNSCC ultimately benefit from CPI treatment, highlighting the need for effective biomarkers for patient selection for these interventions (Ferris et al., 2016; Burtness et al., 2019). Principally, understanding the biological functions and the underpinning mechanisms of these biomarkers could vastly improve the efficacy of immunotherapies (Chen et al., 2019).

Of note is that the combination of high-throughput sequencing technology with bioinformatic analysis provides an efficient approach for the development of biomarkers and the discovery of their underlying mechanisms in different cancers (Langfelder and Horvath, 2008). For instance, the weighted gene co-expression network analysis (WGCNA) algorithm, used for analyzing relationships between gene sets and clinical features, has been widely applied to identify targeted modules and hub genes in cancer networks, such as breast cancer and gastric cancer (Tian et al., 2020; Rezaei et al., 2022). In this study, the RNA sequencing (RNA-Seq) datasets of patients with HNSCC were analyzed and the key genes involved in disease occurrence and development identified by subsequent bioinformatic analysis. To unravel the clinical utility of those potential biomarkers, sufficient diagnostic and prognostic models have been constructed. To examine the underlying biological mechanisms of these potential biomarkers in HNSCC, their expression levels, methylation status, and the degree of immune infiltration were verified by multiple databases and experiments. This study reveals a novel panel of potential prognostic biomarkers that may be involved in HPV-related inflammatory TME remodeling and immune evasion. Our findings could provide a basis for further exploration of the clinical significance and molecular mechanism and for facilitating immunotherapy development for HNSCCs.

## Materials and methods

### Data acquisition and pre-processing

The methodological workflow of the current study is illustrated in **Supplementary Figure S1**, which included data acquisition, processing, and validation. The RNA-Seq data were downloaded from the cohort of The Cancer Genome Atlas (TCGA) Head and Neck Cancer (HNSC) in the UCSC database (https://xenabrowser.net/datapages/), including 502 tumor tissue samples (500 primary site tumors and 2 metastatic tumors) and 44 normal tissue samples. The referenced gene chip dataset GSE138206 (HG-U133_Plus_2; Affymetrix Human Genome U133 Plus 2.0 Array) was downloaded from the Gene Expression Omnibus (GEO) database (https://www.ncbi.nlm.nih.gov/geo/), including six oral squamous cell carcinoma (OSCC) tumor tissue samples and six paired normal tissue samples. The filterByExpr function of the edgeR package was performed to eliminate low-expression samples from the RNA-Seq data (Robinson et al., 2010). The RMA algorithm of the Affy package was used for background correction and standardization (log2 transformation) of the chip datasets (Gautier et al., 2004). Raw data (counts) from the TCGA dataset were standardized by TPM (transcript per million) before WGCNA.

### Identification of differentially expressed genes

This study used DESeq2, edgeR, and limma to screen the RNA-Seq data and microarray datasets and subsequently fish out the differentially expressed genes (DEGs) between the HNSCC and normal tissue samples (Love et al., 2014; Ritchie et al., 2015). A |log2foldchange| > 1 and *p*_.adjust_ < 0.01 were adopted as the analysis thresholds.

### Construction of the weighted gene co-expression network in HNSCC

Based on the variability of genes as determined using median absolute deviation (MAD), a weighted gene co-expression network was constructed, which included the top 5,000 genes selected using the R software “WGCNA” package (Langfelder and Horvath, 2008). Initially, a scale-free network was constructed based on the Pearson’s correlation coefficients between genes and the selected appropriate thresholds. Furthermore, a topological overlapping matrix (TOM) was transformed *via* an adjacency matrix, and the system clustering tree was obtained by hierarchical clustering. Finally, gene modules were established using the dynamic pruning algorithm, and the correlation between these modules and the clinical features was determined.

### Functional enrichment analysis

In order to further understand the function of the selected genes, the R software “ClusterProfilter” package was used for Gene Ontology (GO) enrichment analysis. In addition, Kyoto Encyclopedia of Genes and Genomes (KEGG) analysis was performed for the pathway enrichment analysis of DEGs and the modular genes in the co-expression network of HNSCC (Yu et al., 2012). A *p*_.adjust_ < 0.01 [false discovery rate (FDR) corrected] was taken as the threshold to determine significant GO biological processes and biologically important KEGG pathways. The oncogene analysis platform GSCAlite was used for further pathway enrichment analysis of the selected key genes (Liu et al., 2018).

### Protein interaction network analysis, survival analysis, and screening of targeted genes

Protein–protein interaction (PPI) networks can facilitate the identification of hub genes in a network. In this study, the STRING database was referenced to analyze overlapping genes of the DEGs and key module genes. Thereafter, the key candidate genes were segregated using the Degree, MCC (Maximal Clique Centrality), MNC (Maximum Neighborhood Component), and EPC (Edge Percolated Component) algorithms of the CytoHubba plug-in in Cytoscape software (Chin et al., 2014). Finally, the R software “Survival” package was utilized for survival analysis of the candidate genes, and a *p* < 0.05 was chosen as the cutoff for potential biomarkers with prognostic value in HNSCC.

### Methylation of key genes and immune cell infiltration analysis

The DNA methylation data were downloaded from the head and neck cancer (HNSC) in the DiseaseMeth 3.0 database, including 530 tumor tissue samples and 50 normal tissue samples. The methylation of key genes was analyzed using the DiseaseMeth 2.0 database, in line with Pearson’s correlation mapping generated by R software (Hinshaw and Shevde, 2019). Moreover, TIMER is a comprehensive resource database for systematic analysis of immune infiltrations, which applies RNA-Seq profile data to detect the immune cell infiltration in different tumor tissues (Li et al., 2017). Therefore, we employed this method to analyze the correlation between the expression levels of key genes and the abundance of immune infiltrates, including B cells, CD8^+^ T cells, CD4^+^ T cells, macrophages, neutrophils, and dendritic cells, in HNSCC (*n* = 522). The gene expression levels representing tumor purity are shown at the far left of the panel.

### Prognostic analysis of key genes in HNSCC

To analyze the prognostic value of the key genes in HNSCC, univariate and multivariate Cox proportional risk regression models were formulated. We then predicted the risk score for each HNSCC patient by stepwise multivariate Cox proportional risk regression analysis. The risk scoring model was constructed using the R software package “Survival” and the “SurvMiner” package (Zhang, 2016). The risk score was calculated as follows: expGene1 *… + expGenen * coefficient (expGene represents the expression level of each key gene, and the coefficient is the corresponding regression coefficient generated by the risk model). Consequently, an overall survival (OS) time curve was plotted, which was based on HNSCC patients with a high or a low risk according to the median risk score. In addition, receiver operating characteristic (ROC) curves for 1-, 3-, and 5-year survival were plotted to verify the specificity and sensitivity of the model (Heagerty et al., 2000). In the prognostic analysis, statistical significance was defined as *p* < 0.05.

### Expression verification of key genes

Multiple databases were searched to verify the expression of the key genes. Of these, GEPIA (Gene Expression Profiling Interactive Analysis) is a cancer data mining tool with resources from the TCGA database, the GTEx (Genotype-Tissue Expression) database, and the HPA (Human Protein Atlas) database that include protein expression information in tumor tissue based on immunoassay techniques [Western blotting, immunohistochemistry (IHC), and immunofluorescence] (Tang et al., 2017). In this study, we verified the expression levels of the key genes in tumor and normal tissues *via* the GEO and GEPIA databases. The correlations between the expression levels of key genes and the prognosis of patients with HNSCC were measured in the GEPIA database, and the corresponding protein expression levels of the key genes were verified using the HPA database. A *p* < 0.05 was considered statistically significant.

## Results

### Identification of the differentially expressed genes and enriched biological processes in HNSCC

**Figure 1:**
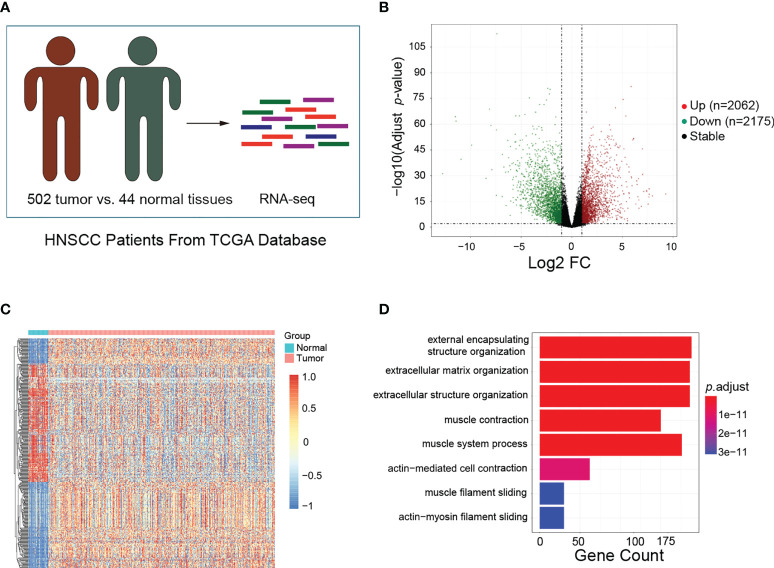
Identification of differentially expressed genes (DEGs) and enriched biological processes in head and neck squamous cell carcinoma (HNSCC). **(A)** Schematic of the patient samples included in the RNA sequencing (RNA-Seq). **(B)** Volcano plots showing the fold change (FC) and *p*-values for the comparisons of tumor tissues and normal tissues. Upregulated (log2FC ≥ 1, *p* < 0.05) and downregulated (log2FC ≤ −1, *p* < 0.05) genes are depicted in *red* and *green*, respectively. **(C)** Top 200 DEGs between tumor and normal tissues visualized as heatmaps from RNA-Seq. Data were *z*-score normalized. **(D)** Gene Ontology enrichment analysis for the top 200 DEGs between tumor and normal tissues. Only the significant enrichment values (*p* < 0.05) of the GO terms from the biological process category are listed.

To identify the DEGs between HNSCC and normal tissues, the transcriptome data obtained from the TCGA database, which included 502 HNSCC and 44 normal tissues, were analyzed (**Figure 1A**). A total of 4,237 DEGs were screened out, including 2,062 upregulated and 2,175 downregulated genes (**Figure 1B**). The top 200 DEGs were used to generate a clustering heat map, in which clear segregation of the gene expression profiles was observed between HNSCC and normal samples (**Figure 1C**). Subsequently, we conducted GO enrichment analysis on the 2,062 upregulated genes to identify the enriched biological processes in HNSCC. Biological processes such as external encapsulating structure organization, external matrix, extracellular matrix, and extracellular structure were found to be significantly enriched in HNSCC (**Figure 1D**). These findings suggest that these DEGs may be involved in TME formation during the occurrence and development of HNSCC.

### Establishing co-expression network modules for HNSCC occurrence and progression

**Figure 2:**
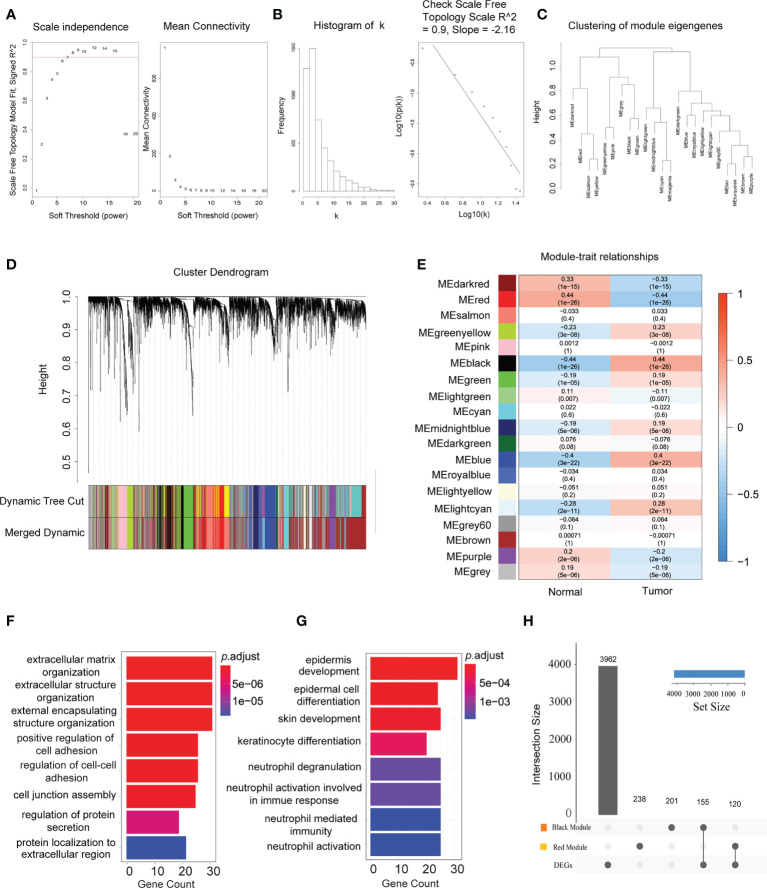
Establishment of the co-expression network modules for the occurrence and progression of head and neck squamous cell carcinoma (HNSCC). **(A)** Scale independence and mean connectivity of the network in different statistical thresholds. The panel displays the strong correlation of the statistical thresholds with a scale-free fit index. **(B)** Robust influence of the statistical threshold on mean connectivity. **(C)** Clustering of the eigengene modules by weighted gene co-expression network analysis (WGCNA) and their correlation with the occurrence and progression of HNSCC. **(D)** Cluster dendrogram based on the dissimilarity of the topological overlap matrix. *Different colours* correspond to the co-expression modules in HNSCC. **(E)** Heatmap of the correlation between the identified modules and clinical features (*n* = 44 in normal tissues and *n* = 502 in tumor tissues). The *x*-axis corresponds to the clinical features, while the *y*-axis represents the identified modules. The *colour scale* (*blue* to *red*) indicates correlation. Data are presented as the correlation coefficient in the *top row* and the *p*-value in the *bottom row in parentheses*. **(F)** Significant enriched Gene Ontology (GO) biological process terms for the HNSCC positively associated (*black*) module. **(G)** Significant enriched GO biological process terms for the HNSCC negatively associated (*red*) module. **(H)** Intersection of the differentially expressed genes in the different modules. *Bar charts* indicate the number of involved genes in the *black* or *red* modules.

Based on the median deviation of the gene expression levels in HNSCC, the top 5,000 genes were selected depending on their ranking of absolute median difference in gene expression levels for the construction of a weighted gene co-expression network. A total of 546 individuals were included to construct the hierarchical clustering map. As illustrated by the results in **Supplementary Figure S2**, there were no obvious outlier samples. With *β* = 6 as the soft threshold to establish the gene scale-free network, a strong topological fitting index was achieved with *R* ^2^ > 0.9 (**Figures 2A, B** **)**. By adopting MEDissThres at 0.25 for merging similar gene modules, the co-expression network was successfully divided into 23 modules (**Figure 2C**). Finally, 19 gene modules were obtained and a module cluster dendrogram was drawn (**Figure 2D**), excluding the genes that could not be classified into other modules for subsequent analysis. Additionally, the association between these 19 modules and the clinical features of patients with HNSCC was assessed and a module–trait relationship diagram depicted (**Figure 2E**). The black module was positively associated with HNSCC, while the red module showed a negative association (correlation coefficients of 0.44 and −0.44 for the black and red modules, respectively, *p* < 0.01). Thus, the black and red modules were selected as the key modules in the occurrence and development of HNSCC. In the additional GO biological process enrichment analysis, the black module mainly included extracellular matrix tissue, extracellular structure tissue, external matrix tissue, and positive regulation of cell adhesion, while the red module corresponded to epidermal development, epidermal cell differentiation, skin development, and keratinocyte differentiation (**Figures 2F, G** **)**. These results indicate that the HNSCC-associated DEGs were mainly concerned with TME remodeling, and overlapping results of the DEGs and key module genes were selected as candidate genes for further analysis (**Figure 2H**).

### Identification and validation of the biomarkers in HNSCC

**Figure 3:**
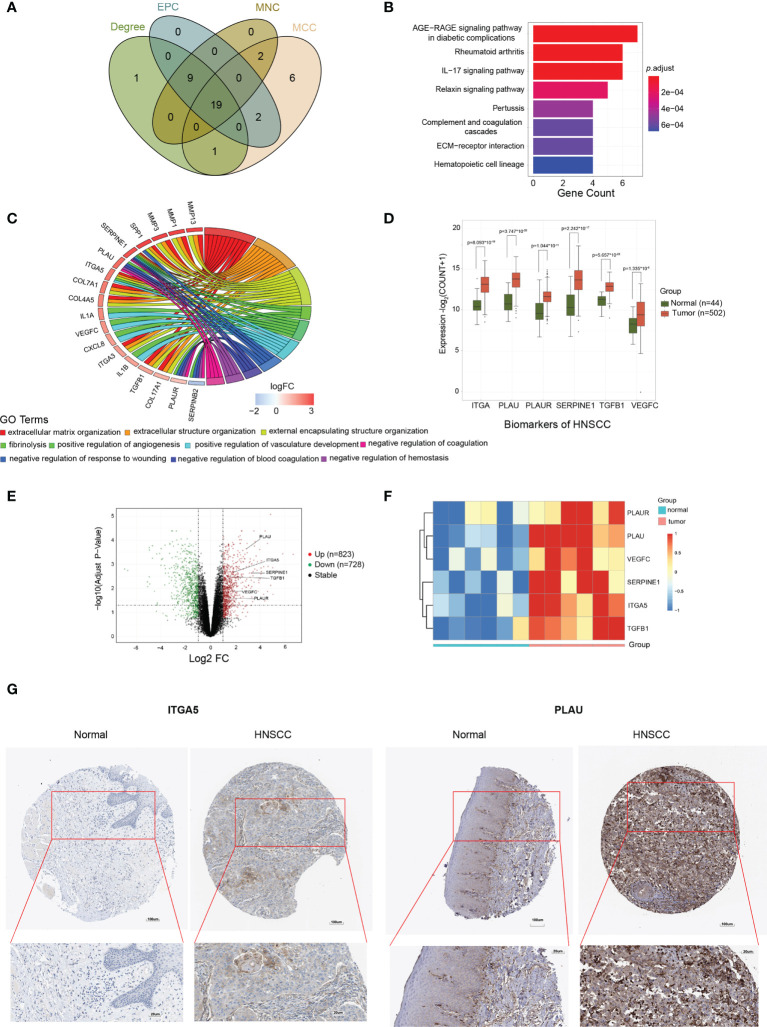
Identification and validation of the biomarkers for head and neck squamous cell carcinoma (HNSCC). **(A)** Venn diagram of the top 30 HNSCC-associated candidates selected by Cytoscape analysis, depending on the MCC (Maximal Clique Centrality), MNC (Maximum Neighborhood Component), EPC (Edge Percolated Component), and Degree algorithms. **(B)** Significant enriched Gene Ontology (GO) pathway signaling for the 19 potential biomarkers. **(C)** Significant enriched GO biological process terms for the 19 potential biomarkers. **(D)** Expression levels of *ITGA5*, *PLAU*, *PLAUR*, *SERPINE1*, *TGFB1*, and *VEGFC* in tumor *versus* normal tissues. **(E)** Volcano plots showing the fold change (FC) and *p*-value of the biomarker candidates for the comparisons of tumor and normal tissues from the GSE138206 dataset. Upregulated (log2FC ≥ 2, *p* < 0.05) and downregulated (log2FC ≤ −2, *p* < 0.05) genes are depicted in *red* and *green*, respectively. **(F)** Expression patterns of *PLAUR*, *PLAU*, *VEGFC*, *SERPINE1*, *ITGA5*, and *TGFB1* in oral squamous cell carcinoma (OSCC) tumor (*n* = 6) and normal (*n* = 6) tissues visualized as heatmaps from the microarray. Data were *z*-score normalized. **(G)** Representative immunohistochemistry staining images of the ITGA5 and PLAU protein expression levels in tumor and normal tissues. Data were from the Human Protein Atlas database.

The above candidate genes were subsequently investigated using the STRING online database. A total of 275 candidates (143 upregulated and 132 downregulated genes) with interaction scores >0.4 were screened out to construct a PPI network, which included 275 nodes and 209,665 connections (**Supplementary Figure S3**). The PPI files were then imported into Cytoscape software, and the CytoHubba plug-in was used to further analyze potential biomarkers in these candidate genes. For reduction of error, the MCC, MNC, EPC, and Degree algorithms were employed. The top 30 candidates with the highest scores were eventually selected (**Supplementary Table S1**), and the overlapping nodes from the different methods used were identified as potential biomarkers of HNSCC. A total of 19 potential biomarkers were identified, namely, *MMP13*, *LCN2*, *COL4A5*, *ITGA3*, *CXCL8*, *TGFB1*, *ITGA5*, *IL1A*, *COL7A1*, *MMP3*, *SERPINE1*, *IL1B*, *SPP1*, *SERPINB2*, *PLAU*, *VEGFC*, *MMP1*, *COL17A1*, and *PLAUR* (**Figure 3A**). To verify the robustness of these screening results, an additional GO enrichment analysis was executed. The results showed that these biomarkers were enriched in the extracellular matrix, extracellular structure, and external matrix tissues in terms of biological processes (**Figures 3B, C** **)**, which are consistent with the enrichment analysis results of the DEGs and the black module. Of these genes, *ITGA5*, *PLAU*, *PLAUR*, *SERPINE1*, *TGFB1*, and *VEGFC* were significantly highly expressed in tumor compared to normal tissues (**Figure 3D**). We validated these potential biomarkers in an independent dataset from the GEO database, which recruited tumor tissues from OSCC that accounts for the high mortality and morbidity rates in HNSCC and paired normal tissues (*n* = 6 for each group) (**Figure 3E**). As expected, *ITGA5*, *PLAU*, *PLAUR*, *SERPINE1*, *TGFB1*, and *VEGFC* were consistently overexpressed in tumor tissues compared to normal tissues (log2FC > |1| and *p* ≤ 0.01) (**Figure 3F**). In addition, increased expressions of the PLAU and ITGA5 proteins in HNSCC tissues compared to normal tissues were observed in the immunohistochemical staining data found in the HPA database (**Figure 3G**). To address the localization of those markers in HNSCC, we explored the HPA database and analyzed the immunohistochemical staining data available for the other markers. The results showed that the ITGA5 and SERPINE1 proteins were mainly expressed in HNSCC tumor cells (with medium staining), while the PLAU protein was enriched in the extracellular matrix of HNSCC (with strong positive reaction). In contrast, the PLAUR and TGFB1 proteins were expressed in tumor cells, but only at a very weak level (data not shown). Unfortunately, there was no valid IHC imaging for the VEGFC protein in HNSCC. Overall, these results further confirmed the validity of the expression levels of the biomarker panel from the perspective of multiple databases.

### Prognostic value of the biomarkers in HNSCC

**Figure 4:**
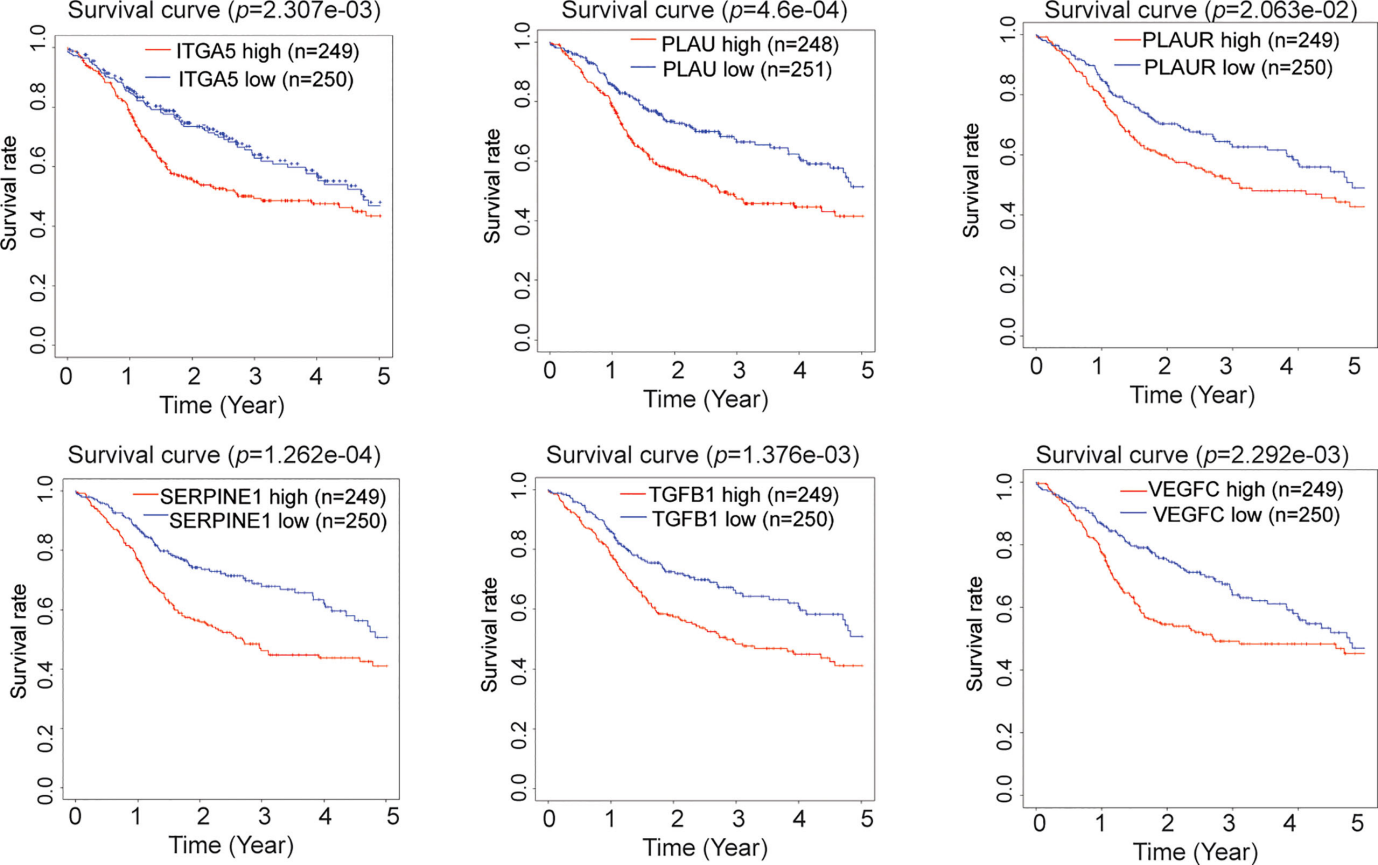
Prognostic value of the biomarkers for head and neck squamous cell carcinoma (HNSCC). Kaplan–Meier survival curves of patients with HNSCC based on the high or low expression levels (above or below median expression) of *ITGA5*, *PLAU*, *PLAUR*, *SERPINE1*, *TGFB1*, and *VEGFC*.

To explore the clinical value of potential biomarkers, Kaplan–Meier analysis was conducted to compare the impact on OS between the high (above median) and the low (below median) expression level of each marker. The results highlighted that *ITGA5*, *PLAU*, *PLAUR*, *SERPINE1*, *TGFB1*, and *VEGFC* presented significant differences on patients’ survival time, revealing that a high expression, rather than a low expression, of these markers correlated with a significantly poorer survival of patients with HNSCC (**Figure 4**). Additionally, these results were verified in the GEPIA database (**Supplementary Figures S4, S5**). Therefore, these six tumor-related genes (*ITGA5*, *PLAU*, *PLAUR*, *SERPINE1*, *TGFB1*, and *VEGFC*) were considered as prognostic biomarkers for HNSCC.

### Prognostic and prediction model construction of the biomarkers in HNSCC

**Figure 5:**
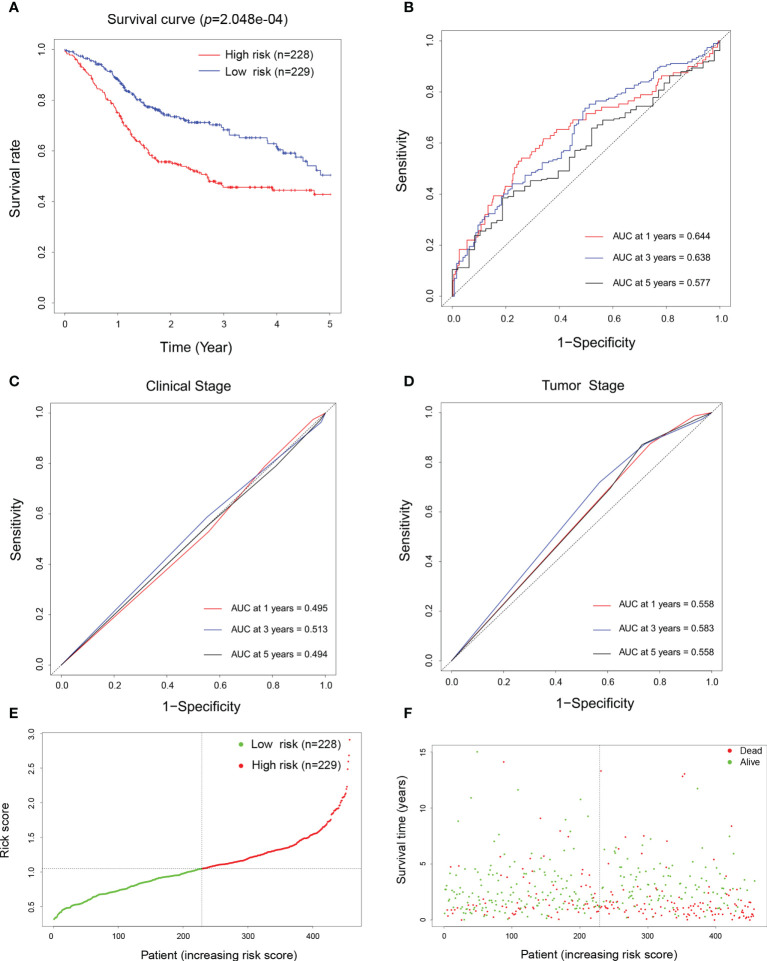
Prognostic and prediction model construction of the biomarkers for head and neck squamous cell carcinoma (HNSCC). **(A)** Overall survival curve of patients with HNSCC depending on their high (*n* = 228) and low (*n* = 229) risk scores defined by the prognostic model. **(B–D)** Diagnostic receiver operating characteristic (ROC) curves generated using the risk scores **(B)**, the TNM clinical staging system **(C)**, and pathological staging system **(D)** aiming to predict the 1-, 3-, and 5-year survival of patients with HNSCC. **(E)** Normalized distribution of the risk scores and the cutoff value for classifying the high- (*red curve*, *n* = 228) and low-risk (*green curve*, *n* = 229) groups. **(F)** Distribution of the survival status of individual HNSCC patients (*red dots* represent dead and *green dots* represent alive) according to their risk scores (*dashed line* represents the cutoff value).

To determine the impact of these biomarker candidates on the prognosis of patients, 457 patients were recruited for prognostic analysis. An OS curve was plotted for the high-risk and low-risk groups (**Figure 5A**), which showed that patients’ survival time was significantly lower in the high-risk group than that in the low-risk group (*p* < 0.001). Furthermore, to validate the usefulness and exemplify the advantage of our prediction models in HNSCC patients’ 1-, 3-, and 5-year outcomes, diagnostic ROC curves were plotted according to their risk scores. Surprisingly, the corresponding area under the curve (AUC) values were 0.644, 0.638, and 0.577, respectively (**Figure 5B**), which were significantly higher than those of the TNM clinical staging system (*p* = 0.0016) and the pathological tumor staging models (*p* = 0.0056) (**Figures 5C, D**, respectively). These results suggest that the current established patient score model has better performance in predicting the OS of patients with HNSCC. Finally, we also examined the distribution of the risk scores and OS of HNSCC patients, which also confirmed the accuracy of this model (**Figures 5E, F** **)**.

### The expression levels of the biomarker candidates are regulated by DNA methylation in HNSCC

**Figure 6:**
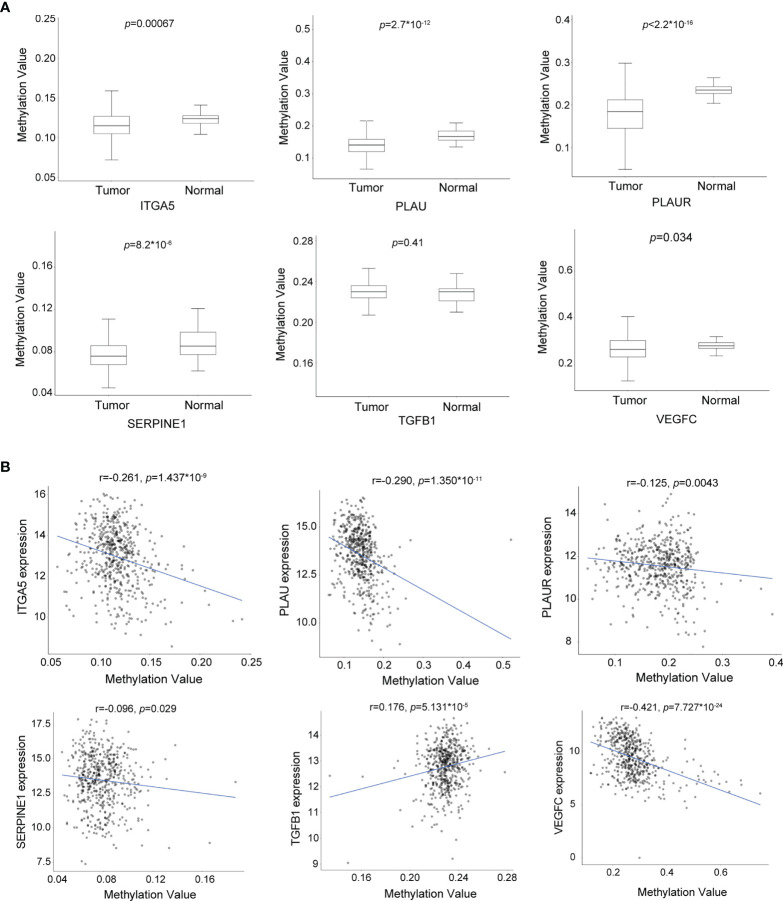
The expression levels of the biomarker candidates are regulated by DNA methylation in head and neck squamous cell carcinoma (HNSCC). **(A)** DNA methylation levels of *ITGA5*, *PLAU*, *PLAUR*, *SERPINE1*, *TGFB1*, and *VEGFC* in HNSCC (*n* = 530) and normal (*n* = 50) tissues. **(B)** Correlation between the DNA methylation levels and gene expression levels of *ITGA5*, *PLAU*, *PLAUR*, *SERPINE1*, *TGFB1*, and *VEGFC* (*n* = 502 and *n* = 22 for HNSCC and normal tissues, respectively). * means multiplication.

To explore the potential mechanism regulating the expression of these biomarker candidates, we analyzed the methylation status of these encoding genes in HNSCC by applying data derived from the Illumina Human Methylation 450 platform. Interestingly, the results demonstrated that, with the exception of *TGFB1*, the DNA methylation levels of the CpG sites in the other five biomarker candidates in tumor tissues were significantly lower in HNSCC tissues (**Figure 6A**). On the other hand, the expression levels of these genes were negatively correlated with DNA methylation (**Figure 6B**), indicating a correlation between the increased gene expression and DNA hypomethylation.

### Biomarkers are involved in TME remodeling and immune infiltration in HNSCC

**Figure 7:**
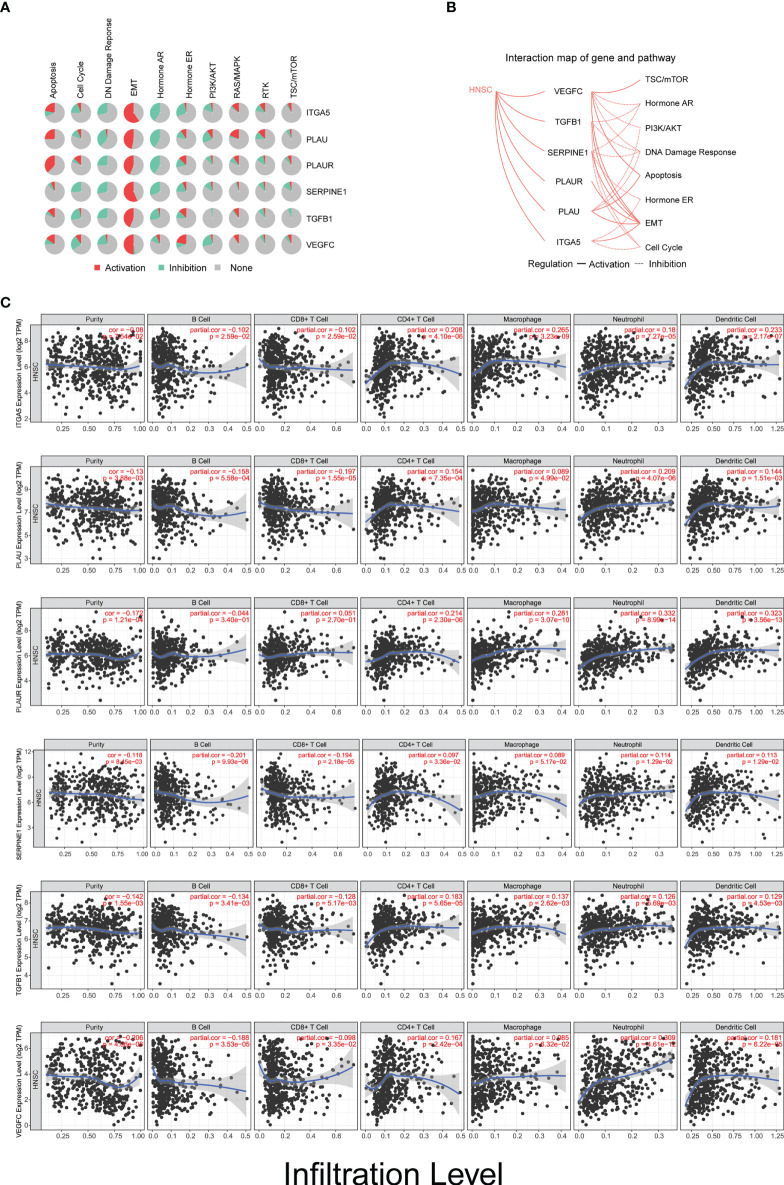
Biomarkers are involved in tumor microenvironment (TME) remodeling and immune infiltration in head and neck squamous cell carcinoma (HNSCC). **(A)** Activation/inhibition functionality of *ITGA5*, *PLAU*, *PLAUR*, *SERPINE1*, *TGFB1*, and *VEGFC* on different biological signaling pathways. **(B)** Interaction map of *ITGA5*, *PLAU*, *PLAUR*, *SERPINE1*, *TGFB1*, and *VEGFC* with potential biological signaling pathways in HNSCC. **(C)** Relationship between the infiltration levels of six types of immune cells and the expression level of the six biomarkers in patients with HNSCC (*n* = 522).

Strikingly, all of these biomarkers are profoundly involved in the activation of the epithelial–mesenchymal transition (EMT) pathway (**Figures 7A, B** **)**. These novel findings indicate that this panel of biomarkers plays a crucial role in TME remodeling and may also contribute to the immune evasion process in the occurrence and development of HNSCC. In order to characterize the underlying biological function of these markers in TME remodeling and immune infiltration, we performed the TIMER algorithm to analyze the correlation between their expression levels and the immune cell infiltration status in HNSCC. Our data showed that the expression levels of these biomarkers in HNSCC were positively correlated with the immune invasion levels of CD4^+^ T cells, macrophages, neutrophils, and dendritic cells, but negatively correlated with the infiltration of B cells. However, for CD8^+^ T cells, with the exception of *PLAUR*, the expression levels of the other five genes were negatively correlated with the infiltrating levels (**Figure 7C**).

### Biomarkers are involved in the transformation and progression of HNSCCs in patients with HPV infection

**Figure 8:**
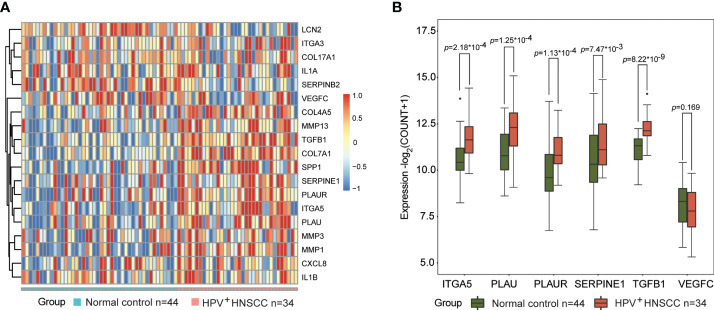
Biomarkers are involved in the transformation and progression of head and neck squamous cell carcinoma (HNSCCs) in patients with human papillomavirus (HPV) infection. **(A)** Expression patterns of 19 differentially expressed genes (DEGs) in HPV-positive HNSCCs (*n* = 34) and normal controls (*n* = 44) visualized as heatmaps. **(B)** Expression levels of *ITGA5*, *PLAU*, *PLAUR*, *SERPINE1*, *TGFB1*, and *VEGFC* in HPV-positive HNSCCs (*n* = 34) and normal tissues (*n* = 44). * means multiplication.

To elucidate the role of these potential biomarkers in HPV-related HNSCC, we analyzed the expression levels of 19 DEGs and 6 biomarker candidates in 34 HPV^+^ HNSCC patients and 44 normal controls. A clear separation of the expression patterns of the 19 DEGs between the two groups (**Figure 8A**) was observed, in which 12 DEGs—*ITGA5*, *SPP1*, *PLAU*, *PLAUR*, *MMP13*, *LCN2*, *COL4A5*, *TGFB1*, *COL7A1*, *SERPINE1*, *SERPINB2*, and *MMP1*—presented statistical significance. More importantly, five out of six biomarker candidates, namely, *ITGA5*, *PLAU*, *PLAUR*, *SERPINE1*, and *TGFB1*, were significantly overexpressed in HPV^+^ HNSCCs compared to normal controls (**Figure 8B**). These results indicate that these biomarker candidates are potentially involved in HPV infection-related transformation and/or malignant progression of HNSCCs, which might be used as early indicators for the development and progression of HNSCCs in patients with HPV infection.

## Discussion

Head and neck cancer is the sixth most common cancer type worldwide, and its incidence and mortality are on a rising trend (Bray et al., 2018; Chow, 2020). Due to the lack of an effective method for early diagnosis, most patients with HNSCC are in the mid to the advanced stages when they present in clinics (Mydlarz et al., 2010); moreover, precise prognosis and predictive biomarkers are also lacking at present (Pignon et al., 2009). Notably, the double-stranded DNA virus HPV infection is one of the major oncogenic factors for HNSCC, and the incidence of HPV-associated HNSCCs has significantly increased in the last few decades (Simard et al., 2012; Ellington et al., 2020). Despite their histological similarities, HPV^+^ and HPV^−^ HNSCCs are now considered as two distinct cancers, owing to their substantial molecular and clinical differences (Leemans et al., 2018). However, due to a lack of effective biomarkers, the clinical management of these diseases is still the same, which leads to unfavorable outcomes and various side effects (Johnson et al., 2020; Lechner et al., 2022). Thus, exploration of clinically applicable biomarkers for HNSCC and an understanding of their underlying molecular mechanisms are urgently needed.

With the continuous development of high-throughput sequencing, gene microarray technologies, and bioinformatics methods, the association between genomic alterations and disease development can be examined at the molecular level, providing great advantages for studying the pathogenesis, diagnosis, and prognosis of diseases (Li et al., 2017; Tang et al., 2017; Liu et al., 2018; Rezaei et al., 2022). Nonetheless, previous studies have tended to focus on an individual gene associated with HNSCC, but cross-validations between different databases have been inadequate (Arroyo-Solera et al., 2019; Fan et al., 2019). In this study, based on HNSCC datasets from the TCGA database, we screened out a group of 19 candidate key genes using different bioinformatic analysis approaches. Subsequent PPI analysis was conducted to build interaction networks among them, followed by GO analysis of the key candidate genes, which showed that they were mainly related to the extracellular matrix, extracellular structure, and external matrix. These microenvironmental components are essential in regulating cellular function and maintaining normal homeostasis (Theocharis et al., 2016). Dysregulation of these components causes microenvironment remodeling, leading to abnormal cell proliferation, invasion, and differentiation, which play important roles during the occurrence and development of many cancers (Lu et al., 2011; Caon et al., 2020).

Indeed, there are two predominant reasons contributing to HPV^+^ HNSCC development: i) genetic alterations of host cells are exerted by the integration of viral DNA into the host genome, which causes genomic instability, as well as a transcriptional shift by oncoviral proteins E6 and E7 that interacts and destabilizes a large number of host proteins (Leemans et al., 2018); ii) HPV infection breaks the dynamic and complex network of intercellular communication, which leads to an oncogenic TME remodeling (Gameiro et al., 2022). However, the key genes involved in the aforementioned processes and their biological functions are not well defined. Here, we finally identified five candidates—*ITGA5*, *TGFB1*, *PLAU*, *PLAUR*, and *SERPINE1*—as potential biomarkers for the prognosis and diagnosis of HPV-related HNSCC, which may play essential roles in the initiation and development of HNSCC. Additional pathway analysis highlighted that these biomarker candidates are particularly involved in EMT transformation, which is a cancer-specific biological process in which epithelial cells lose their cellular stickiness and polarity and transform from a non-motile epithelial cell to a mesenchymal cell signature, acquiring characteristics of migration and invasion (Thiery, 2002; Tsai and Yang, 2013).

Of note is that the current study identified a novel panel of biomarker candidates for HNSCC diagnosis and prognosis, which have yet to be sophisticatedly studied in relation to EMT and immunomodulation, especially with HPV-related HNSCC. To the best of our knowledge, with the exception of *TGFB1*, which has been proposed to be correlated with the clinical outcomes of HPV^+^ oropharynx squamous cell carcinoma patients after radiotherapy (Tao et al., 2018), none of the other genes have been previously reported with regard to HNSCC development and patient prognosis. Several studies have suggested that carcinoma cells activate SNAIL signaling and secrete large quantities of TGFB1 protein, together with other immunoregulatory cytokines and chemokines to accelerate EMT transformation (Wrzesinski et al., 2007; Teicher, 2007; Scheel et al., 2011), which act as immunosuppressive factors that induce regulatory T cells (Tregs) and attenuate the cytolytic activities of natural killer (NK) cells (Bellone et al., 1995; Viel et al., 2016). Thus, *TGFB1* blockades have been considered to have great promise for enhanced antitumor activity; hence, anti-*TGFB1* therapeutics, such as antisense oligonucleotide-, ligand-, and receptor-targeted neutralizing antibodies, and small molecule inhibitors were broadly tested in different cancer types (Akhurst and Hata, 2012; Colak and ten Dijke, 2017). Recently, a preclinical study has demonstrated that the combination of galunisertib (a small molecule inhibitor of TGFB1 protein receptor I kinase) with an anti-PD-L1 (programmed death-ligand 1) regime presented a thoroughly improved antitumor effect compared to either galunisertib or anti-PD-L1 monotherapy, which may be attributed to the induction of enhanced antitumor T-cell activities by galunisertib (Holmgaard et al., 2018). Therefore, examination of the potential function in TME remodeling and immunomodulation of the rest of the markers in the panel were of paramount interest.

Intriguingly, the expression levels of the biomarker candidates, including *ITGA5*, *PLAU*, *PLAUR*, *SERPINE1*, and *VEGFC*, were positively correlated with the infiltration of CD4^+^ T cells, macrophages, neutrophils, and dendritic cells, but negatively correlated with the infiltration of B cells in HNSCC. However, for the infiltrating levels of CD8^+^ T cells, except for no significant association found for *PLAUR*, the other markers showed negative correlation. Markedly, in patients with HPV^+^ HNSCC, lymphocyte infiltration is generally associated with improved prognosis (Solomon et al., 2018). After adjusting the effect of HPV infection, HNSCC tumors with higher T cell (especially for CD8^+^ T cells) infiltrates were found to be associated with significantly better survival (Keck et al., 2015; Matlung et al., 2016). In contrast, the infiltration of CD4^+^ T cells (particularly Tregs) plays an immunosuppressive role, which led to decreased CD8^+^/Treg ratios within tumors in spite of the high CD8^+^ T-cell infiltrates, and is correlated with poor prognosis of patients with HNSCC (Russell et al., 2013; Mandal et al., 2016). In addition, macrophages recruited to the immunosuppressive TME would transform into tumor-associated macrophages, which secrete the immunosuppressive cytokines, including IL-1beta, IL-6, IL-10, TGFB1, and the PD-L1 protein and other checkpoint ligands, which are correlated with unfavorable outcomes for patients with HNSCC (Marcus et al., 2004; Costa et al., 2013). Of note is that the TGFB1 protein expression has been shown to be positively correlated with HPV infection in cervical lesions (Iancu et al., 2010). To date, the association between other immune cell infiltration status and HNSCC patient survival remains to be determined, and further studies are warranted to understand the function of this panel. High-risk HPV infection has been established as a risk factor of developing HNSCC. In general, HPV-related HNSCC is believed to exhibit increased immune infiltrates and improved response to anti-PD-1 therapy compared to HPV-unrelated tumors (Partlová et al., 2015; Mandal et al., 2016; Chen et al., 2018; Wang et al., 2019). However, the composition of immune cells in the tumor immune microenvironment (TIME) is quite complex and heterogeneous. Thus, more specific biomarkers that can be used to characterize the TIME in HNSCCs are needed. More importantly, exploring the biological interactions of HPV infection and these biomarker candidates will help researchers have a better understanding of HPV-related HNSCC development. In this study, we identified five genes, namely, *ITGA5*, *TGFB1*, *PLAU*, *PLAUR*, and *SERPINE1*, as biomarker candidates involved in TIME remodeling for HPV-related HNSCCs. To this end, attenuating the expression of these identified candidate genes in tumors can potentially enhance the infiltration of CD8^+^ T cells and B cells and decrease the infiltration of CD4^+^ T cells and macrophages, which would be a promising direction for improving patient outcomes and developing targeted therapeutics for HNSCC.

In conclusion, with sophisticated bioinformatic analysis of transcriptome data from cancer and normal tissues, we have identified a novel panel of DEGs for HPV-related HNSCC, which included *ITGA5*, *PLAU*, *PLAUR*, *SERPINE1*, and *TGFB1*. Following verification and validation of the expression levels and clinical impact of these candidate genes in multiple databases, we propose that this panel of candidate genes could potentially be used as a biomarker panel in the prognostic evaluation and early detection of HPV-related HNSCC. Furthermore, these key players are likely to be involved in TME remodeling and immune evasion, which affect the response to immune therapy for HNSCC. Our work reveals predictors/biomarkers for improved immune therapy response in HPV-related HNSCC.

## Data availability statement

Publicly available datasets were analyzed in this study. The data analyzed during this study are available at The Cancer Genome Atlas (TCGA; https://cancergenome.nih.gov/) and the Gene Expression Omnibus (GEO) database (https://www.ncbi.nlm.nih.gov/geo/).

## Author contributions

QZ, CW, and HC conceived the original idea and conceptualized the study design, which were refined by OY, GX, and HZ. HC and TH collected the data for the study, which were analyzed and interpretated together with QZ, OY, and JW. QZ, OY, and HC drafted the manuscript, which was revised by JW, HZ, and CW. JW, HZ, and CW supervised the study. All authors critically reviewed and finalized the manuscript and gave their approval for submission.

## Funding

This study was supported by grants from the National Natural Science Foundation of China (No.82103658 and No.82072287) and the Shanghai Pujiang Program (No.22PJD040).

## Acknowledgments

We thank Peihan Guo for valuable scientific discussions. Aaron Chen-Xiao, from Peninsula High School at Rancho Palos Verdes, CA, USA, is acknowledged for data analysis. Andrew C. McCourt is acknowledged for revising the manuscript.

## Conflict of interest

The authors declare that the research was conducted in the absence of any commercial or financial relationships that could be construed as a potential conflict of interest.

The reviewer WuZ declared a shared affiliation with authors QZ and OY, to the handling editor at the time of review.

## Publisher’s note

All claims expressed in this article are solely those of the authors and do not necessarily represent those of their affiliated organizations, or those of the publisher, the editors and the reviewers. Any product that may be evaluated in this article, or claim that may be made by its manufacturer, is not guaranteed or endorsed by the publisher.
