# Supplementary material for: Bioinformatic analysis identifies HPV-related tumor microenvironment remodeling prognostic biomarkers in head and neck squamous cell carcinoma
Source: Front Cell Infect Microbiol. 2022 Nov 8;12:1007950. doi: 10.3389/fcimb.2022.1007950 (PMC9679011; doi:10.3389/fcimb.2022.1007950)
Supplement: Supplementary file 1 [file DataSheet_1.docx]

Supplementary Material

## Supplementary Figures

##
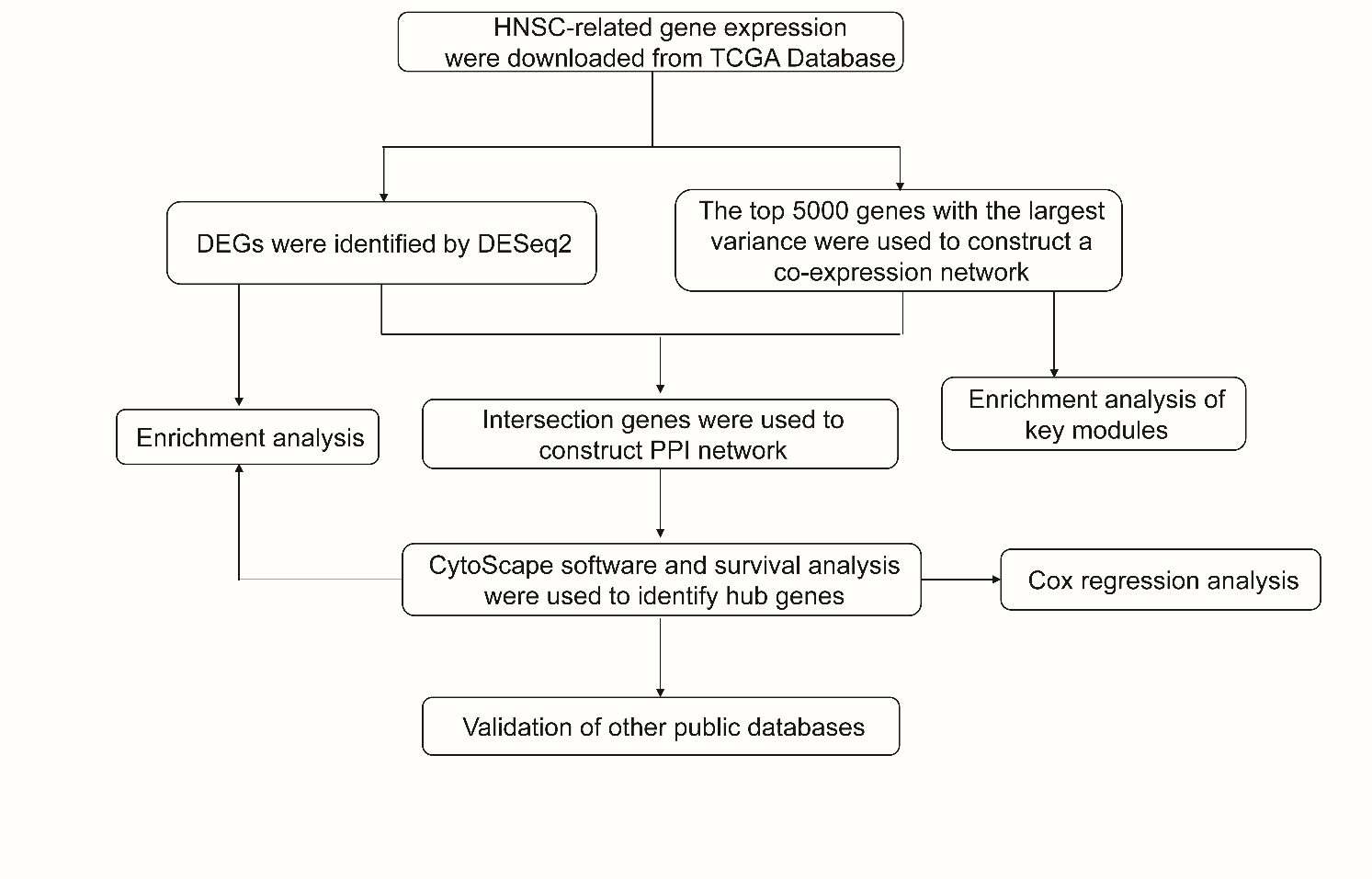
Supplementary Figure S1. Methodological workflow of the current study.

**

**

## Supplementary Figure S2. A cluster dendrogram containing 546 individuals that no obvious outlier samples were presented.


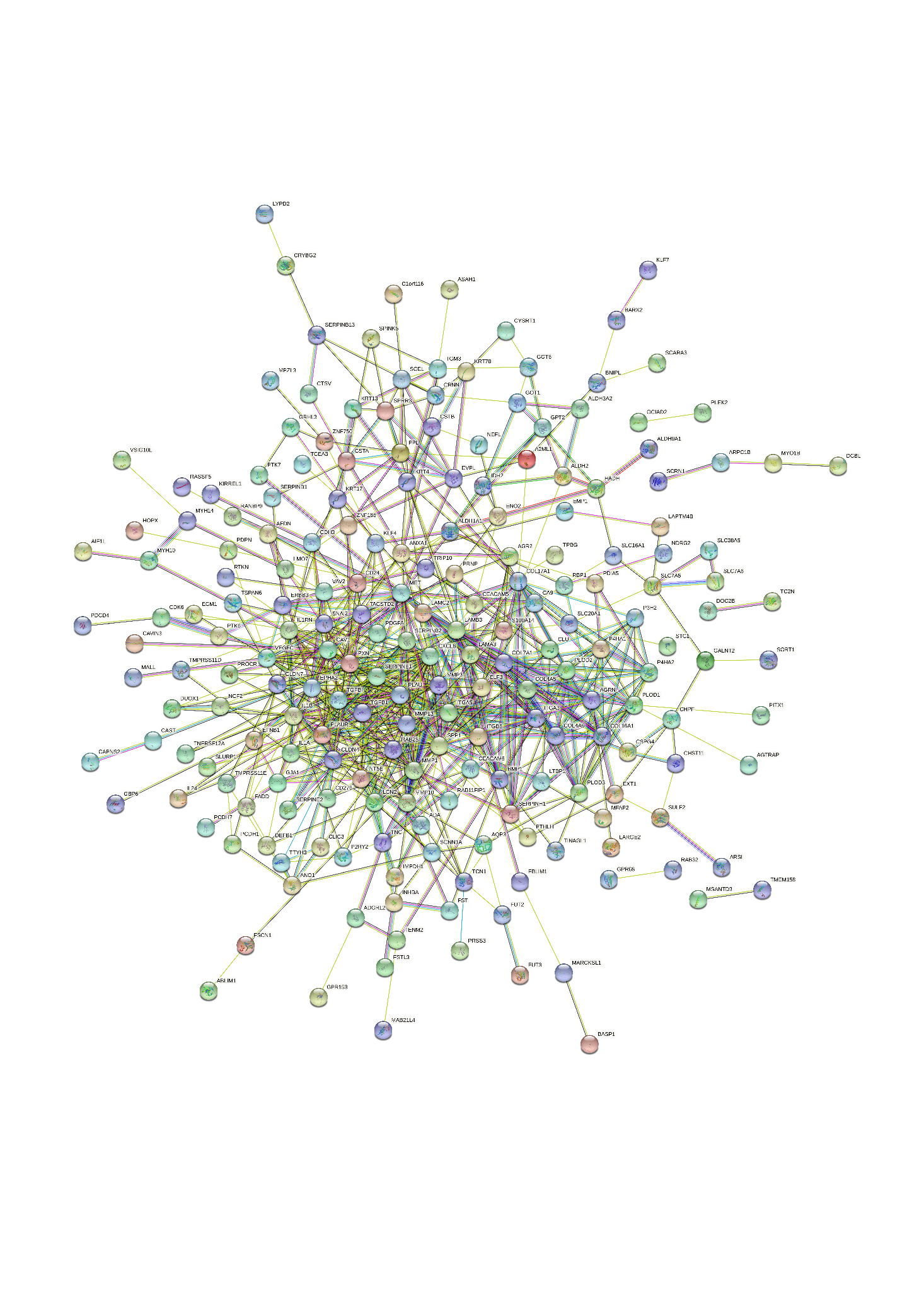


**Supplementary Figure S3.** STRING analysis result that containing 275 genes and 209,665 connections among them.


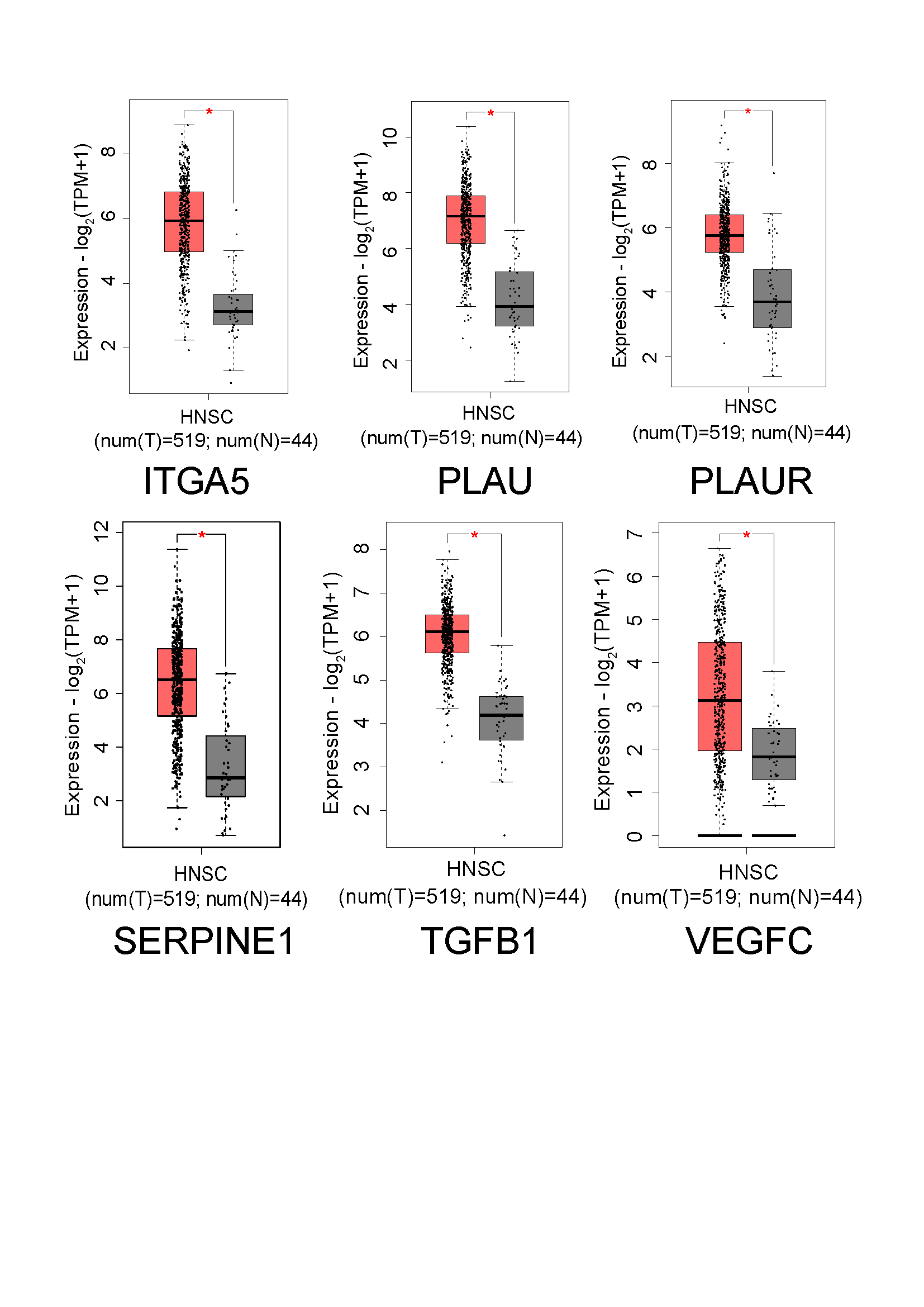


**Supplementary Figure S4.** Expression levels of ITGA5, PLAU, PLAUR, SERPINE1, TGFB1 and VEGFC in tumour and normal tissue (data acquired from the GEPIA database).


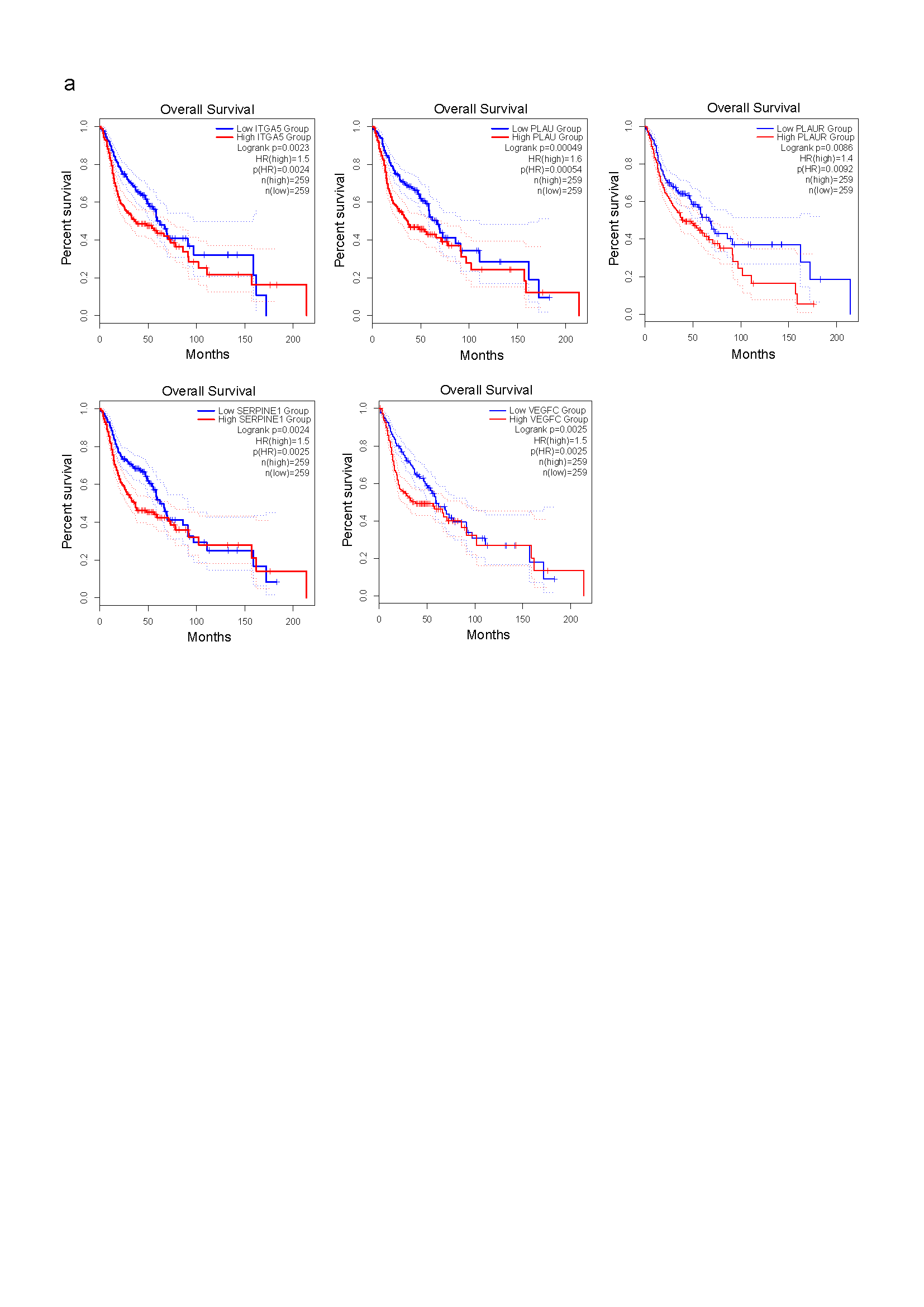


**Supplementary Figure S5.** Verification of expression levels of potential biomarkers (ITGA5, PLAU, PLAUR, SERPINE1, TGFB1 and VEGFC) on HNSCC patient survival via GEPIA database.

Supplementary Table S1. The top 30 hub genes identified by the four methods in CytoHubba

| Category | Rank Methods in CytoHubba | | | |
| --- | --- | --- | --- | --- |
|  | Degree | EPC | MCC | MNC |
| 1 | IL1B | CXCL8 | CXCL8 | CXCL8 |
| 2 | CXCL8 | SPP1 | SERPINE1 | TGFB1 |
| 3 | TGFB1 | SERPINE1 | IL1B | IL1B |
| 4 | MET | TGFB1 | MMP1 | SPP1 |
| 5 | SERPINE1 | MMP1 | COL4A5 | SERPINE1 |
| 6 | SPP1 | MMP3 | COL7A1 | MMP1 |
| 7 | MMP1 | IL1B | COL16A1 | MET |
| 8 | CAV1 | MET | PLOD1 | MMP3 |
| 9 | ITGA5 | CAV1 | PLOD2 | CAV1 |
| 10 | PXN | PLAU | P4HA1 | ITGA5 |
| 11 | MMP3 | ITGA5 | P4HA2 | PLAU |
| 12 | ITGA3 | ITGA3 | MMP3 | ITGA3 |
| 13 | LAMB3 | MMP13 | TGFB1 | LAMA3 |
| 14 | PLAU | LAMA3 | IL1A | COL7A1 |
| 15 | COL17A1 | LAMB3 | PLOD3 | LAMC2 |
| 16 | LAMC2 | LAMC2 | COL4A6 | LAMB3 |
| 17 | LAMA3 | PLAUR | COL17A1 | COL4A5 |
| 18 | COL7A1 | PXN | SPP1 | PXN |
| 19 | SNAI2 | VEGFC | LCN2 | SNAI2 |
| 20 | COL4A5 | IL1A | IL1RN | MMP13 |
| 21 | MMP13 | COL7A1 | P3H2 | COL17A1 |
| 22 | CLDN4 | ITGB6 | SERPINB2 | PLAUR |
| 23 | SERPINB2 | MMP10 | PLAU | LCN2 |
| 24 | ITGB6 | COL4A5 | MMP13 | IL1A |
| 25 | VEGFC | SERPINB2 | MMP10 | NT5E |
| 26 | PLAUR | SNAI2 | PLAUR | SERPINB2 |
| 27 | LCN2 | LCN2 | SERPINH1 | ITGB6 |
| 28 | IL1A | COL17A1 | VEGFC | VEGFC |
| 29 | NT5E | NT5E | ITGA5 | COL4A6 |
| 30 | PLOD1 | IL1RN | ITGA3 | COL16A1 |
